# Early androgen exposure and human gender development

**DOI:** 10.1186/s13293-015-0022-1

**Published:** 2015-02-26

**Authors:** Melissa Hines, Mihaela Constantinescu, Debra Spencer

**Affiliations:** Department of Psychology, University of Cambridge, Cambridge, UK

**Keywords:** Androgen, Testosterone, Amniotic, Human, Behavior, Gender, Sex, Play, Mini-puberty, Autism

## Abstract

During early development, testosterone plays an important role in sexual differentiation of the mammalian brain and has enduring influences on behavior. Testosterone exerts these influences at times when the testes are active, as evidenced by higher concentrations of testosterone in developing male than in developing female animals. This article critically reviews the available evidence regarding influences of testosterone on human gender-related development. In humans, testosterone is elevated in males from about weeks 8 to 24 of gestation and then again during early postnatal development. Individuals exposed to atypical concentrations of testosterone or other androgenic hormones prenatally, for example, because of genetic conditions or because their mothers were prescribed hormones during pregnancy, have been consistently found to show increased male-typical juvenile play behavior, alterations in sexual orientation and gender identity (the sense of self as male or female), and increased tendencies to engage in physically aggressive behavior. Studies of other behavioral outcomes following dramatic androgen abnormality prenatally are either too small in their numbers or too inconsistent in their results, to provide similarly conclusive evidence. Studies relating normal variability in testosterone prenatally to subsequent gender-related behavior have produced largely inconsistent results or have yet to be independently replicated. For studies of prenatal exposures in typically developing individuals, testosterone has been measured in single samples of maternal blood or amniotic fluid. These techniques may not be sufficiently powerful to consistently detect influences of testosterone on behavior, particularly in the relatively small samples that have generally been studied. The postnatal surge in testosterone in male infants, sometimes called mini-puberty, may provide a more accessible opportunity for measuring early androgen exposure during typical development. This approach has recently begun to be used, with some promising results relating testosterone during the first few months of postnatal life to later gender-typical play behavior. In replicating and extending these findings, it may be important to assess testosterone when it is maximal (months 1 to 2 postnatal) and to take advantage of the increased reliability afforded by repeated sampling.

## Review

This review critically evaluates the available evidence regarding the impact of two early surges of testicular hormones on the development of human behaviors and psychological characteristics that differ by sex. The review begins by summarizing the large body of experimental research in non-human animals that documents the influences of testosterone on mammalian neurobehavioral development. These studies form the basis for predicting that testosterone influences human neurobehavioral development, and their findings provide guidance as to the types of effects that would be hypothesized to occur in humans. The next section of the review critically evaluates research investigating the role of testosterone in the development of human behavior. Because testosterone would be expected to influence behaviors that show sex differences, this section begins by reviewing evidence as to which human behaviors show sex differences and discusses the sizes of the differences. Similarly, because testosterone would be expected to influence human sexual differentiation at times when it differs in males and females, this section next summarizes information as to when these times occur. The section also discusses methodological approaches to studying possible influences of testosterone on human development, given that proper experiments, where individuals are randomly assigned to be treated with testosterone or placebo early in life, are not ethically acceptable. The evidence that testosterone influences human neurobehavioral development prenatally or neonatally is then evaluated. The review concludes by summarizing various approaches and their pitfalls, by providing guidance for evaluating the robustness of studies relating early testosterone to gender-related outcomes and by suggesting that the early postnatal surge in testosterone, sometimes called mini-puberty, might be particularly accessible for studying the role of testosterone in human development.

### Gonadal hormone influences on neurobehavioral development in non-human mammals

Thousands of studies of non-human mammals have documented the contribution of gonadal steroids, particularly the testicular hormone, testosterone, to sexual differentiation of the brain and of behavior [1]. Female animals treated with testosterone prenatally or neonatally subsequently show increased male-typical behavior and decreased female-typical behavior. Similarly, male animals that have their testes removed early in development later show increased female-typical behavior and reduced male-typical behavior. These effects have been seen for sexual behaviors and for non-reproductive behaviors that differ by sex, such as aggression and juvenile rough-and-tumble play [2]. They also have been seen in numerous species ranging from rodents to non-human primates [1,2].

Early hormone manipulations produce changes in the mammalian brain as well as in behavior. The sexually dimorphic nucleus of the preoptic area (SDN-POA) provides a prototypical example of these effects. The SDN-POA is larger in volume and contains more neurons in male than in female rats. Treating female rats with testosterone during early development increases SDN-POA volume in adulthood and castrating developing males reduces it [3]. Testosterone and hormones produced from it have been found to influence cell survival, neurite outgrowth, and neurochemical specification during early development in rodents [4]. The effects of early androgen exposure on both the brain and behavior are often described as organizational, and early androgen exposure is thought to produce enduring changes in behavior by altering the development and organization of underlying brain circuits.

Several general principles have emerged from the thousands of studies of androgenic influences on development in non-human mammals, and these principles can guide studies evaluating the possibility of similar influences on the human brain and on human behavior. First, the brain regions and behaviors that are influenced show sex differences, meaning that they differ on the average for male and female animals. Therefore, only those human behaviors that show sex differences would be expected to relate to early androgen exposure. Second, the periods when testosterone influences development correspond to times when testosterone is higher in developing male than in developing female animals. Therefore, investigating testosterone in humans at times when it shows dramatic sex differences provides the best opportunity to see any effects that might exist. Third, testosterone can contribute to individual differences within each sex in gender-related characteristics, as well as to differences between the sexes, and the influences of testosterone are generally linear and graded, with increasing doses of testosterone producing increasingly large effects. Treatment outside the physiological range can sometimes produce non-linear effects, perhaps because of feedback mechanisms that reduce endogenous hormone production or because of full receptor occupation. Thus, when studying humans, the influences of testosterone within the physiological range would be expected to be linear, with higher concentrations of testosterone relating to more male-typical, or less female-typical, behavior. Outside the physiological range, testosterone exposure might have no effect, however, or even the reverse effect to that seen at lower concentrations.

### Evaluating the human evidence

Before beginning the review of human research, it is useful to define some terms, point out some potential pitfalls in studying human gender-related behavior, and discuss some current concerns about the reliability of findings in the biomedical sciences generally. We will use the terms “gender difference” and “sex difference” interchangeably to describe the average differences in the behavior of males and females. We will also sometimes use the terms “behavior” and “psychological characteristics” interchangeably, to describe one or the other of these, or both. Regarding potential pitfalls in studying gender, one is the tendency to analyze findings by gender, even when there was no initial hypothesis about gender differences [5]. This tendency, combined with the tendency to publish significant, but not insignificant, findings can lead to an over-reporting of gender differences. A second potential pitfall relates to correlational analyses. When two characteristics each show a gender difference, they will often correlate significantly in mixed groups of males and females, simply because both characteristics differ by gender. Therefore, in correlational studies, it is important to analyze data within each sex, rather than only in males and females combined. Regarding concerns about research reliability, flexibility in analytic approaches and data inclusion/exclusion practices allows virtually any data set to produce statistically significant results [6], and it has been suggested that most published research findings in the biomedical sciences are not replicable or are false [7]. Therefore, this review will point out which findings have been independently replicated and which have not. Attention also will be drawn to findings when they differ from those reported by others and when they are inconsistent with what would be hypothesized based on the experimental findings in other species.

#### Which human behaviors show average sex differences?

There have been numerous meta-analyses of human behavioral sex differences. A review of these meta-analyses concluded that men and women and boys and girls generally behave similarly, with most behaviors showing negligible or small gender differences [8]. There are some notable areas of difference, however. In all cases, distributions for males and females overlap, but for some behaviors, males and females differ more substantially than for others [9]. The sizes of the differences in specific characteristics can be expressed in standard deviation units. To put these units in a familiar context, the sex difference in height is two standard deviation units in size. Some psychological sex differences are larger than the sex difference in height, but most are smaller. For instance, the tendency to have a primary erotic attraction to females is many times as large in males as in females (*d* = 5.0 to 6.0), as is the sex difference in identifying with the male gender (*d* > 10.0), and both of these sex differences are substantially larger than the sex difference in height. In contrast, sex differences in personality characteristics, such as empathy or physical aggression, and in cognitive abilities, such as mental rotations or verbal fluency, are smaller. For instance, the largest well-established cognitive sex difference is that seen in performance accuracy on a specific three-dimensional (3D) mental rotations task (*d* = 0.9, favoring males); whereas the sex difference in verbal fluency is relatively small (*d* = 0.3, favoring females). Sex differences on specific measures of personality characteristics, such as empathy (higher in females than in males) and aggression (higher in males than in females) also range from about *d* = 0.2 to *d* = 1.0. Sex differences in childhood play behavior, such as preferences for toys like vehicles and dolls or responses on inventories measuring a range of gender-typed play activities, can be as large or somewhat larger than the sex difference in height (*d* = 1.8 to 2.8). (See [2,9,10] for more detailed reviews of the sizes of specific behavioral and psychological gender differences). In the broader context of any type of group difference in psychological characteristics, differences of half a standard deviation (*d* = 0.5) are considered to be of moderate size, those of 0.8 or greater are considered to be large, and those of about 0.2 are considered to be small. Differences smaller than 0.2 are viewed as negligible [11]. For correlations, *r* values of 0.5 are considered to be large, those of 0.3 are considered to moderate, and those of 0.1 are considered to be small.

In addition to average differences in some aspects of the behavior of males and females, there are sex differences in the likelihood of being diagnosed with certain psychiatric disorders [12]. Males are more likely than females to be diagnosed with autism spectrum disorders (ASD), with Tourette syndrome and other tic-related disorders, with attention deficit/hyperactivity disorder (ADHD) and with conduct disorder. In contrast, from adolescence on, females are more likely than males to be diagnosed with depression. Females also are more likely than males to be diagnosed with eating disorders.

#### When during early development, does testosterone differ in males and females?

**Figure 1:**
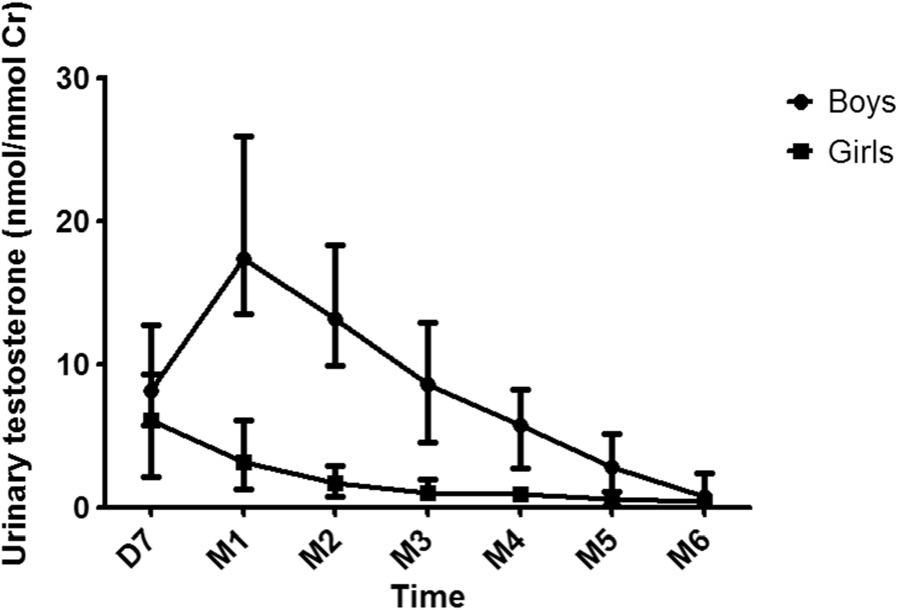
**Median urinary testosterone concentrations in male and female infants from 7 days to 6 months of age.** Error bars represent the interquartile range. Testosterone was measured at day 7 postnatal (D7) and then monthly from 1 month (M1) to 6 months (M6) of age. Urinary testosterone was measured using HPLC-tandem mass spectrometry. Urinary testosterone concentrations are higher in boys than in girls, and testosterone peaks in boys at 1 month of age, is lower before and after this age, and declines to baseline by about age 6 months. The figure is reproduced from [78] by permission of Elsevier.

Testosterone is notably higher in males than in females during two periods of early human development - from about weeks 8 to 24 of gestation - and during the first few months after birth [13-15]. These, therefore, are the times when testosterone is likely to influence human gender development. In addition, the sex difference in testosterone in infants appears to be largest at about the first to the second month of postnatal life and to be smaller before and after that, reaching baseline by about 6 months of age [13-15] (also see Figure 1).

#### How can hormonal influences on human development be studied?

It generally is unethical to manipulate testosterone during early human development for experimental purposes. Instead, information has come from individuals who developed in unusual hormone environments, including individuals with genetic conditions, individuals whose mothers were prescribed hormones during pregnancy, and boys who were reassigned as girls early in life, because of penile damage. A second approach has been to relate early testosterone concentrations in typically developing individuals to later gender-related behavior. Studies also have related the ratio of the second to the fourth digit of the hand to sex-typical behavior, on the assumption that this digit ratio reflects prenatal androgen exposure. The relationship of finger ratios to prenatal androgen exposure appears to be too weak to be useful in studies of typically developing individuals, however, and studies relating finger ratios to sex-typed behavior have produced inconsistent results [9,16,17]. Consequently, studies using finger ratios to assess prenatal androgen exposure are not included in this review.

#### Evidence from individuals exposed to unusual hormone environments prenatally

The strongest evidence that prenatal androgen exposure contributes to human gender development comes from studies of children’s gender-typed play. Girls exposed to elevated androgens prenatally, because they have the genetic condition, congenital adrenal hyperplasia (CAH), show increased male-typical toy, playmate, and activity preferences, a finding replicated in over a dozen studies from several independent research groups in several different countries, including the United States, the United Kingdom, Sweden, and Germany [18].

Because these results are not from controlled experimental studies, it is important to consider whether the behavioral differences between girls with and without CAH might result from factors related to the CAH condition other than prenatal androgen exposure. For instance, children with CAH experience reduced corticosteroids prenatally and girls with CAH are born with some degree of genital ambiguity, caused by their prenatal androgen exposure. Children with CAH are treated with corticosteroids postnatally to normalize their hormone levels. Behavioral masculinization in girls with CAH is unlikely to be caused by corticosteroid abnormality or treatment, however, because boys with CAH have similar corticosteroid abnormality and treatment (although their androgen levels appear to be largely normal prenatally), but they do not show increased behavioral masculinization. Evidence that healthy children whose mothers were prescribed androgenic progestins during pregnancy show increased male-typical play, and that those whose mothers were prescribed anti-androgenic hormones show reduced male-typical play [19-22], also suggests that androgen exposure, rather than other aspects of the CAH condition, are responsible for the behavioral differences seen in girls with CAH. Similarly, data from individuals with another genetic condition, complete androgen insensitivity syndrome (CAIS), also suggest a contribution of early androgen exposure to male-typical play behavior. These XY individuals have normally functioning testes but are born with feminine-appearing external genitalia, because their cells are unable to respond to the androgens produced by their testes. Adults with CAIS recall engaging in reduced male-typical, and increased female-typical, play behavior in childhood [23], and these effects have been replicated by an independent research group for children with CAIS as well [24]. Thus, several lines of evidence converge on the conclusion that prenatal exposure to androgenic hormones increases male-typical play behavior in children. This convergent evidence is important, because it suggests that the common factor across the different study populations, androgen exposure, is responsible for the behavioral outcomes, rather than the other consequences of the individual situations (e.g., corticosteroid abnormality, ambiguous external genitalia) that are not shared across populations.

Women exposed to elevated androgens prenatally because of CAH also are less likely to be exclusively or almost exclusively heterosexual than are other women, a finding that has again been independently replicated cross-nationally [25-28]. Across studies, the majority of women with CAH are exclusively or almost exclusively heterosexual, but about 30% are not, a figure markedly higher than the about 5% or fewer in the general population. Individuals with CAIS almost always have a sexual orientation toward men, consistent with their lack of effective androgen exposure, and this finding also has been reported by more than one independent research group [23,29]. Sexual orientation also has been reported for two male infants who were reassigned as girls because of severe penile damage in infancy. One of these individuals had primary sexual interest in women in adulthood, whereas the other had sexual interest in both women and men [30,31]. Both outcomes are consistent with an influence of early androgen exposure on sexual orientation.

In regard to gender identity, or one’s sense of self as male or female, exposure to high levels of androgens prenatally has been linked to an increased likelihood of developing a male gender identity, despite being reared as a female [32]. A change to living as a man has been observed in about 1% to 3% of women with CAH, as well as in women with other genetic conditions causing exposure to elevated concentrations of androgens during early development [33-36], and again has been seen cross-nationally. The strength of identification as female also seems to be reduced in women and girls with CAH, even when they do not wish to change to live as males [28,37,38]. There also is evidence from more than one research group that individuals with CAIS almost always develop a female gender identity, consistent with their lack of effective androgen exposure [23,29,39]. Male infants reassigned as girls following early damage to their penis have also been followed up for gender identity. In one such individual, gender identity was male, despite female sex of rearing from 18 months of age [30]. In the other individual, gender identity was female, following female sex of rearing from 7 months of age [31]. This last finding suggests powerful influences of the social environment on gender identity, given the female gender identity with female sex of rearing, despite male-typical early androgen exposure and a Y chromosome. It is important to note, however, that this finding is not only a single, as yet unreplicated, report, but that it involves a single individual.

Evidence linking early androgen exposure to behaviors other than sex-typed childhood play, sexual orientation, and gender identity is not as strong, in most cases, because fewer studies have been conducted. Two independent research groups have reported that females with CAH show increased physical aggression, however [40-42]. Although earlier reports had suggested no increase in fighting in girls with CAH [43,44], these reports were based on small samples and used weak measures (e.g., unspecified or single interview questions). Evidence from boys and girls exposed to androgenic progestins prenatally, because their mothers were prescribed these hormones during pregnancy, also suggests a link between prenatal androgen exposure and later physically aggressive behavior [45], but this finding has not been independently replicated. It also is unusual in finding that boys, as well as girls, show increased aggression following prenatal exposure to androgenic progestins.

In regard to cognitive abilities linked to gender, reports to date are inconsistent. For spatial abilities, two studies have found that girls or women with CAH perform better on measures of mental rotations [46,47], but several others have not [48-51]. Sample size and measurement reliability could explain some of the inconsistencies, but the study using the largest sample and the measure that shows the largest sex difference [49] did not find that females with CAH performed better than unaffected females on mental rotations. Similarly, one study found that females with CAH performed worse than controls on a verbal fluency task [50], but similar results were not found in a second study [48]. There is even less evidence regarding links between early androgen exposure and other gender-related behaviors. One study found decreased empathy in females with CAH [40] and another found increased autistic traits [52], but these findings await independent replication attempts.

#### Evidence from studies of normal variability in testosterone prenatally

Studies of normal variability in testosterone prenatally include studies that have measured testosterone in maternal blood during pregnancy and studies that have measured testosterone in amniotic fluid. These studies provide some evidence suggesting that normal variability in prenatal testosterone exposure may relate to later gender-typical behavior, although none of the findings have yet been independently replicated.

Regarding testosterone in maternal blood, one study found that maternal testosterone at mid-pregnancy positively predicted male-typical play behavior at 3 and ½ years of age for girls [53]. A similar relationship was not seen for boys, however. A study from a different research group found that maternal sex hormone-binding globulin (SHBG; a protein which limits the availability of testosterone to act), measured at a similar time during pregnancy, in combination with testosterone in adulthood, positively predicted sex-typed behavior in adult female offspring [54]. Men were not included in this study. We were able to find two studies that investigated the relationship between maternal and fetal testosterone. One study did not find a significant relationship between maternal and fetal testosterone [55], but the second study reported a significant positive correlation between maternal and fetal testosterone (*r* = 0.42) [56].

Regarding testosterone measured in amniotic fluid, one study has reported that testosterone measured in amniotic fluid positively predicts male-typical play in both boys and girls [57]. Two other studies have not found a similar relationship, however [58,59]. It is not clear whether these failures to see the relationship reflect the use of smaller samples or less sensitive measurement tools or whether the relationship between testosterone in amniotic fluid and sex-typical childhood play is weak or non-existent. Other data suggest that amniotic fluid testosterone may not be a reliable measure of fetal androgen exposure. The one study that we have been able to find that related testosterone in amniotic fluid to testosterone in fetal blood did not find a significant correlation [55].

Researchers have also looked at relations between normal variability in amniotic fluid testosterone and performance on spatial tasks. Two studies found no significant correlation between amniotic fluid testosterone and accuracy scores on a mental rotation task in either boys or girls [60,61]. One of the studies [60] is frequently cited as supporting the idea that prenatal testosterone exposure influences spatial ability, and so it merits a closer look. The measure of mental rotations used in the study did not show a statistically significant sex difference in the usual outcome variable, accuracy. In addition, the authors subdivided their samples of girls and boys into those who appeared to be using a rotational approach and those who did not, in an exploratory fashion, and reported findings only for these four subgroups, ranging in size from 7 to 13 children, and not for the full groups of 21 girls and 20 boys. Also, they looked at three performance variables, in addition to accuracy, variables for which there was no prior evidence of gender differences. Significant findings included a positive correlation between amniotic fluid testosterone and speed of rotation among girls who were judged to be using a rotational strategy, but a negative relationship in boys who were judged to be using a rotational strategy. These are the findings that are typically cited to support an effect of early androgen exposure on spatial abilities. The findings are in opposite directions; however, and neither finding would have been predicted based on previously reported patterns of sex differences or on sex differences in the study itself, since speed of rotation did not show a sex difference. A different research group has reported a significant correlation between amniotic fluid testosterone and performance on an embedded figure task [61]. The large sex difference seen on the task in this study (*d* = 1.15) contrasts with prior meta-analytic research [62,63] showing small sex differences on this type of task. As noted above, this study also found no correlation between amniotic fluid testosterone and performance on a mental rotation task. This negative result occurred even though the mental rotation task showed a sex difference of reasonable size (d = 0.6).

One research group also has looked at the correlation between amniotic fluid testosterone and a range of characteristics related to ASD in subgroups from a population of several hundred children for whom testosterone had been measured in amniotic fluid. These studies also merit careful examination, because they are widely cited to support a contribution of early androgen exposure to ASD. Different reports from this group are based on different subsamples from the larger population, with the subsamples ranging in size from about 20 individuals of each sex to about 100 individuals of each sex, depending on the study. An initial study found a correlation between amniotic fluid testosterone and reduced vocabulary in girls and boys combined [64], but both variables showed a sex difference and correlations were not significant within either girls or boys. Thus, the significant finding might simply reflect the existence of a sex difference on each of the two measures. A second study reported a significant curvilinear relation between amniotic fluid testosterone and eye contact in boys but not in girls [65], but this curvilinear relationship, as opposed to a linear relationship, had not been predicted. These studies were part of a larger project that also measured looking preferences for social/non-social stimuli, non-verbal communicative gestures, verbalizations, pretend play, and empathy [66]. None of these other measures related significantly to amniotic fluid testosterone in either girls or boys. The numerous measures, the small sample size and the single, unpredicted, statistically significant within-sex relationship observed, suggest the possibility of chance findings.

Subsequent studies from the same research group have reported significant relationships between amniotic fluid testosterone and various measures of empathy that show sex differences favoring girls, and on questionnaires assessing restricted interests, systemizing, and characteristics related to autism, on which boys typically score higher than girls. One study found a significant negative correlation between amniotic fluid testosterone and scores on the Child Version of the Empathizing Quotient in boys but not in girls [67]. This study also found negative correlations between amniotic fluid testosterone and performance on a measure of judging emotions in eyes, for boys and for girls. A second study found no significant correlation in either boys or girls between amniotic fluid testosterone and performance on a measure of empathy that involved describing interactions between animated triangles [68]. The same research group also found that amniotic fluid testosterone correlated with restricted interests in boys, though not in girls [69], and with scores on the Systemizing Quotient—Child Version [70] in boys and in girls. Similarly, they have reported significant correlations between amniotic fluid testosterone and scores on the Child Autism Spectrum Quotient and the Quantitative Checklist for Autism in Toddlers in boys and in girls [71,72] and on the Childhood Autism Spectrum Test in boys but not in girls [71]. There have been no reported independent replication attempts, at least as yet, for these findings of relationships between amniotic fluid testosterone and empathy, restricted interests, or scores on measures of characteristics related to ASD.

Most recently, this research group has reported that testosterone measured in amniotic fluid does not relate to a diagnosis of ASD [73]. This study examined testosterone and four other hormones in amniotic fluid for 128 males diagnosed with ASD compared to 217 male controls. Data for girls with and without ASD were not reported. Amniotic fluid testosterone did not differ significantly for males with and without ASD, nor did androstenedione, progesterone, 17 alpha-hydroxy-progesterone, or cortisol. Instead, a principal component, extracted from values for all five hormones, was higher in boys with a diagnosis of ASD. Although these findings do not support suggestions that ASD relates to early testosterone or androgen exposure, they might suggest an alternative mechanism related to hormones, although not one for which there is evidence of a link to gender. These findings also await attempts at independent replication.

Other disorders that show discrepant sex ratios have not been the focus of as much research on possible links to early testosterone exposure as have ASD. Similar ideas have been put forward in regard to tic-related disorders [74] and ADHD [75], however, both of which, like ASD, are more often diagnosed in males than in females. Evidence that individuals diagnosed with tic-related disorders also are more male-typical than might be expected in other areas that have been related to prenatal androgen exposure, e.g., in their childhood play interests, has been suggested to provide some support for prenatal androgenic influences on tic-related disorders. As yet, suggestions that early testosterone exposure relates to ADHD are largely theoretical. Eating disorders are more common in females than in males, and it has been suggested that testosterone exposure during early development might protect against these disorders [76]. Findings of reduced disordered eating in females with male co-twins compared to females with female co-twins have been interpreted to support this hypothesis, based on the assumption that females with male co-twins are exposed to some of their male co-twin’s testosterone [76]. A second team of researchers failed to replicate this finding, however [77], although this replication attempt used a different measure to assess disordered eating.

Thus, although a primary basis for interest in understanding the influences of testosterone on human behavior has been the idea that androgens might contribute to discrepant sex ratios for psychological diagnoses, research in this area has been relatively scant for disorders other than ASD, and for ASD has ultimately produced findings that do not support a contribution from prenatal testosterone exposure. This lack of research, and even the lack of clearly supportive findings, might reflect, in part, the rarity of individuals with both atypical androgen exposure during early development and a gender-linked psychological diagnosis and the lack of a powerful measure of prenatal androgen exposure in individuals without hormone abnormality. The measures of prenatal testosterone exposure that have been used in typically developing individuals, including measurements of testosterone in amniotic fluid, and comparing individuals with male versus female co-twins, may not be powerful enough to detect effects consistently in the sample sizes available.

#### The early postnatal testosterone surge and human behavior

The early postnatal surge in testosterone, sometimes called mini-puberty, may be more accessible than the prenatal surge. One study measured testosterone repeatedly in urine samples from infants beginning at week 1 and continuing each month from weeks 4 to 26 of postnatal life (months 1 to 6 postnatal) (Figure 1). Gender-typical play was then measured using a questionnaire, the Pre-School Activities Inventory (PSAI), and by observing toy choices in a playroom at age 14 months. The area under the curve for testosterone during the first 6 months of life was significantly larger in boys than in girls. In addition, the area under the curve for testosterone positively predicted PSAI scores for male-typical play, negatively predicted observed play with a baby doll in boys, and positively predicted observed play with a train in girls [78]. There have not yet been any attempts at independent replication of these findings.

Other researchers have measured salivary testosterone in infants at the age of 3 to 5 months and measured behavior either on the same day [79,80] or later in life [72]. None of these studies found a significant sex difference in testosterone, perhaps because the samples were taken on only a single occasion when infants were 3 to 5 months of age, after the peak of the early postnatal surge. Testosterone appears to be highest at the first month of postnatal life and to have declined substantially by about the third to fourth month (Figure 1). The single, rather than repeated, measurement might have further reduced the power of these studies. The studies looking at behavior on the same day as saliva sampling have reported some correlations between testosterone and behavior. These correlations involved behaviors that do not show sex differences, however, and so would not be predicted to relate to testosterone. The study that measured behavior later in life found no relationship between testosterone in saliva and scores on the Quantitative Checklist for Autism in Toddlers [72]. The relatively late, single assessment of postnatal testosterone might have contributed to this negative result.

## Conclusions

The relationship of finger ratios to prenatal androgen exposure appears to be too weak to be useful in studies attempting to relate prenatal androgen exposure to later behavior [9,16,17], and this review suggests that this may be the case for testosterone measured in amniotic fluid as well. Although one research group has found predicted relationships, others have not. Small sample sizes may have contributed to the failures to find significant effects, but the insignificant correlation that has been reported between testosterone in fetal blood and testosterone in fetal amniotic fluid [55] also does not support the robustness of the amniotic fluid approach.

Researchers have recently begun to measure testosterone in early infancy, during the early postnatal testosterone surge. This approach to understanding relations between early androgen exposure and human gender development has two advantages. First, in contrast to maternal blood or amniotic fluid, it provides measures from the developing individual him or herself. Second, the postnatal surge can be sampled on repeated occasions and sampling can be controlled for time of day.

So far, behavioral researchers have measured testosterone during infancy in urine or in saliva, and urine measures, but not saliva measures, have shown a sex difference. However, in these studies, saliva samples were obtained on only one occasion, whereas in the study using urine samples, they were obtained repeatedly, on seven occasions between birth and 6 months of age. In addition, saliva samples were obtained only after the peak in testosterone, further limiting the potential to detect sex differences. Repeated sampling between about 4 to 8 weeks of age, at controlled times of day, is likely to provide the best chance of detecting sex differences in testosterone, as well as relationships to later gendered behavior.

Recommendations for future studies aimed at evaluating the influence of early androgen exposure on human behavior echo those described elsewhere [16,81,82]. These recommendations include focusing on behaviors that show sex differences and hypothesizing linear effects, particularly within the physiological range. Recommendations for correlational studies also include analyzing correlations within each sex, rather than in the sexes combined, to reduce spurious findings. It has been argued that most research findings in the biomedical sciences are false [6,7]. The guidelines for conducting research on relationships between testosterone and behavior can also be adapted to help researchers evaluate whether findings in this area are likely to be replicable. Confidence in replicability is increased if a study 1) assesses outcome variables that show sex differences; 2) assesses testosterone at a time when it is higher in developing males than females; 3) looks at relationships in males and females separately; and 4) finds linear relationships within the physiological range. Other more general recommendations for avoiding spurious findings also apply to this area of research, including reporting all groups and all variables that were assessed, and indicating how sample sizes were determined. If this research area is to develop into a more mature science, it will also be important to replicate effects and report both significant and insignificant findings to facilitate meta-analysis and estimation of effect sizes.

## Abbreviations

ASD: Autism spectrum disorder
ADHD: Attention deficit/hyperactivity disorder
CAH: Congenital adrenal hyperplasia
CAIS: Complete androgen insensitivity syndrome
PSAI: Preschool activities inventory
SDN-POA: Sexually dimorphic nucleus of the preoptic area
SHBG: Sex hormone-binding globulin
